# Angiographic and Clinical Impact of Novel Revascularization for Occluded Femoropopliteal Prosthetic Bypass Graft: A Combination of Surgical Thrombectomy and Drug-Coated Balloon Angioplasty

**DOI:** 10.1155/2023/6730220

**Published:** 2023-11-21

**Authors:** Tatsuro Takei, Takashi Kajiya, Keisuke Yamamoto, Junichiro Takaoka, Yoshihiko Atsuchi, Nobuhiko Atsuchi

**Affiliations:** ^1^Department of Cardiology, Tenyoukai Central Hospital, Kagoshima, Japan; ^2^Department of Cardiovascular Surgery, Tenyoukai Central Hospital, Kagoshima, Japan

## Abstract

**Background:**

Previous reports have revealed various endovascular intervention techniques for prosthetic femoropopliteal bypass occlusion (PFPBO); however, treatment for PFPBO remains challenging for most interventionalists and vascular surgeons because the procedure is complicated. Most of the reported techniques involve device implantation. In the present study, we performed a combination of surgical graft thrombectomy and drug-coated balloon angioplasty for PFPBO without implanting any additional devices. Furthermore, we determined the favorable long-term results of this treatment using follow-up angiography. *Case Presentation*. A 77-year-old man with a history of chronic kidney disease and coronary artery disease presented to our clinic with rest pain on his left leg. Seven years prior to the current consult, he underwent femoropopliteal bypass (FPB) surgery using a prosthetic graft due to in-stent occlusion of the left superficial femoral artery (SFA). Four years after surgery, a duplex ultrasound scan revealed stenosis of the proximal anastomosis site; hence, medical therapy was continued. On the current consult, diagnostic angiography revealed occlusion of the FPB and infrapopliteal vessels. In the first attempt at recanalization, the guidewire was unable to pass through the occluded SFA. Therefore, another technique was performed to revascularize the FPBO and infrapopliteal vessels. We obtained an angiography of the left leg after inserting the guiding sheath via the right common femoral artery (CFA). First, surgical thrombectomy using a Fogarty catheter via the exposed left CFA was performed. Following endovascular therapy via the right CFA, we performed drug-coated balloon angioplasty for anastomotic stenosis and recanalized occlusive infrapopliteal vessels. Restenosis was not observed on follow-up angiograms. On further follow-up angiography, there was notable regression of the residual stenosis at the proximal anastomosis of the prosthetic graft.

**Conclusion:**

This novel revascularization strategy may be a viable treatment option for PFPBO.

## 1. Introduction

Prosthetic femoropopliteal bypass grafting has been established as a treatment option for long-occluded superficial femoral arteries (SFA), and long-term results have been determined [1]. Although there are reports of inferior patency rates compared to autologous venous conduits [2], prosthetic femoropopliteal bypass grafting is a necessary treatment modality for patients without a saphenous vein.

Recently, we recognized that the while Viabahn stent graft (Gore, Flagstaff, AZ) is comparable to femoropopliteal bypass (FPB) surgery in the treatment of superficial femoral arterial occlusive disease [3], FPB surgery is also useful in cases where endovascular therapy (EVT) has failed. Furthermore, the effectiveness of FPB, including prosthetic grafting, in the treatment of chronic limb-threatening ischemia (CLTI) has been demonstrated, and open surgery revascularization may become more widespread in the future [4].

Under these circumstances, we often encounter patients with prosthetic femoropopliteal bypass occlusion (PFPBO) in the clinical setting. Maintaining bypass graft patency is crucial in patients with CLTI, and graft occlusion has been reported to be a risk for major amputation of lower extremities [5]. Furthermore, PFPBO may be associated with a higher risk of acute exacerbation of limb ischemia; hence, appropriate revascularization is desirable [6]. Although PFPBO in patients with lower extremity artery disease, including CLTI, are generally treated with a new surgical bypass graft, thrombectomy, thrombolysis, major surgical graft revision, or EVT [7, 8], standardized treatment is yet to be established.

EVT for PFPBO is challenging because occluded prosthetic grafts contain significant thrombi. Interventional procedures for PFPBO have the potential risk of limb-threatening emboli. Furthermore, redo procedures for PFPBO, including surgical graft thrombectomy+transluminal angioplasty and thrombolysis+transluminal angioplasty, have demonstrated unfavorable results, as evidenced by Pedersen et al., who showed that the primary patency rate of these redo procedures was 28% at 6 months and 12% at 12 months [9].

In this study, we report the case of a patient with PFPBO who underwent a combination of surgical graft thrombectomy and drug-coated balloon (DCB) angioplasty. We confirmed the patency of the prosthetic graft on follow-up angiographies until 18-month posttherapy.

## 2. Case Presentation

A 77-year-old man with a history of chronic kidney disease and coronary artery disease was referred to our institution due to rest pain on his left leg. Seven years prior to the current consult, FPB surgery was performed using a Dacron prosthetic graft due to in-stent occlusion of the left SFA. Four years after the procedure, a duplex ultrasound scan revealed proximal anastomosis stenosis; hence, medical therapy was continued.

**Figure 1:**
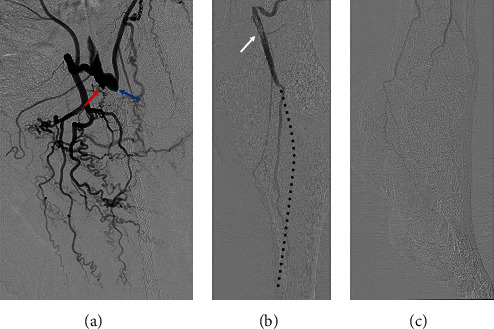
The diagnostic angiography. (a) Red arrow indicates the ostium of the occluded FPB. Blue arrow indicates the ostium of the occluded DFA. (b) Blood flow was observed in the proximal part of the PA via the collateral vessels. A floating thrombus was observed in the proximal part of the PA (white arrow). The middle part of the PA and PTA was occluded (dotted line). (c) Blood flow in the plantar artery through the collateral vessels was observed. DFA: deep femoral artery; FPB: femoropopliteal bypass; PA: popliteal artery; PTA: posterior tibial artery.

Upon current examination, the ABI on the left side was extremely low and unmeasurable. On his right side, the ABI was 1.1, which was within the normal value. A diagnostic angiography of the left lower extremities showed total occlusion of the native SFA, FPB, and ostium at the left deep femoral artery (DFA) (Figure 1(a)). In addition, the angiography revealed a floating thrombus in the proximal popliteal artery (PA) and occlusion from the middle PA to the posterior tibial artery (PTA) (Figure 1(b)). The distal PTA, plantar artery, and peroneal artery were detected via collateral circulation (Figure 1(c)).

In the first attempt at recanalization, we decided to treat the in-stent occlusion of the left SFA using an endovascular approach. Percutaneous cannulation of the right common femoral artery was performed, followed by insertion of a 0.035-inch guidewire, advancement of a 6 Fr sheath, and insertion of the IMA catheter into the left iliac artery, after which an initial angiogram was obtained. We then changed 6 Fr sheath to a 6 Fr destination guiding sheath (Terumo Corporation, Tokyo, Japan) and delivered it to the left common femoral artery (CFA) using a crossover approach. The stent was treated with Halberd (Asahi Institute Corporation, Aichi, Japan), Jupiter DP 60 (Boston Scientific Corporation, MA, USA), and Jupiter MAX (Boston Scientific Corporation, MA, USA) in an antegrade manner but was unable to advance into the in-stent occlusion. Despite using the looped wire technique with a 0.035-inch Radifocus guidewire (Terumo, Tokyo, Japan), it was not possible to pass through the proximal cap of the occlusion owing to the hardened nature of the lesion.

A series of procedures were performed to prepare for another attempt at recanalization. A Halberd guidewire (Asahi Inc.) was advanced into the occluded FPB, and we assessed the lesion using intravascular ultrasound (IVUS) and Eagle Eye Platinum ST (Philips, Amsterdam, The Netherlands). IVUS findings showed proximal and distal anastomosis stenosis and prosthetic graft occlusion filled with a significant thrombus. The stenotic lesion at the proximal anastomosis site was dilated with a 5 × 40 mm JADE balloon (OrbusNeich, Hong Kong).

**Figure 2:**
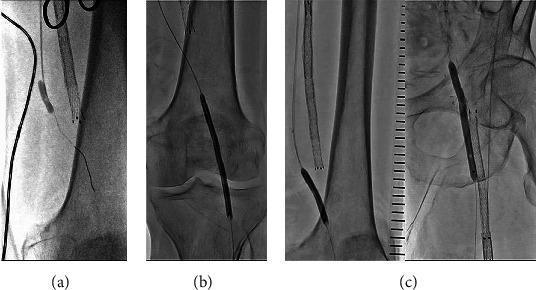
(a) A Fogarty catheter was used to remove the thrombus in the graft. (b) DCB angioplasty using a 4 × 80 mm IN.PACT Admiral was performed on the popliteal artery. (c) DCB angioplasty using a 5 × 40 mm IN.PACT Admiral was performed on the distal anastomosis. DCB angioplasty using a 6 × 80 mm IN.PACT Admiral was performed on the proximal anastomosis. DCB: drug-coated balloon.

As the guidewire did not pass through the in-stent occlusion in the initial revascularization attempt, resulting in its failure, we decided to treat PFPBO with a combination of surgical thrombectomy and endovascular intervention under general anesthesia in the second attempt at revascularization. A 6 Fr destination guiding sheath (Terumo, Tokyo, Japan) was advanced to the left CFA using a crossover approach, and preoperative angiogram of the left lower extremity was obtained. The left CFA was exposed by the cardiovascular surgeons using a cut-down approach. We then advanced a 300 cm Gladius MG guidewire (Asahi Institute Corporation, Aichi, Japan) via an occluded FPB to the middle PA from the exposed left CFA. We performed a Fogarty catheter thrombectomy (Edwards Lifesciences, Irvine, CA, USA) and removed the thrombus from the occluded FPB and proximal PA (Figure 2(a)). After thrombectomy, angiography revealed reperfusion of the FPB and moderate stenosis at both anastomosis sites.

**Figure 3:**
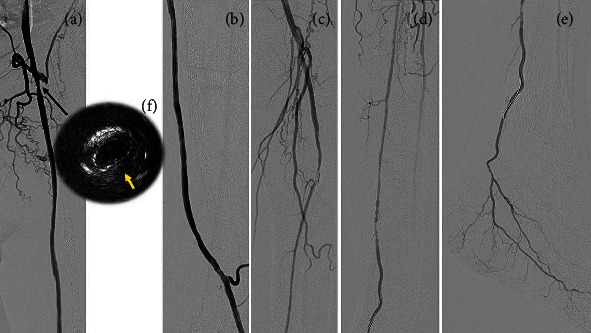
Angiograms immediately after therapy (a–e). (f) IVUS findings immediately after treatment of the proximal anastomosis. Moderate stenosis due to isoechoic plaque (yellow arrow) was noted. IVUS: intravascular ultrasound.

Subsequently, we performed EVT for anastomotic stenosis on the occluded PA and PTA. Unfortunately, the Gladius MG was unable to pass through the occluded PA in an antegrade manner. Thereafter, retrograde wiring was performed via PTA. The distal PTA was punctured with a 20-gauge needle, and retrograde guidewire passage was achieved using a Halberd guidewire with a Prominent Advance NEO 2 microcatheter (Tokai Medical Products, Aichi, Japan). Once the microcatheter was advanced to the proximal popliteal artery, the retrograde guidewire was changed to a 300 cm Gladius MG guidewire. A 120 cm GOGO catheter (Medikit, Tokyo, Japan) was advanced deeply into the PA via the right CFA. Wire externalization was achieved by inserting a retrograde wire into the GOGO catheter, after which a 3 × 100 mm RapidCross (Medtronic, MN, USA) was used to dilate the occluded lesions from the middle PA to the proximal PTA. We performed DCB angioplasty for the middle PA using a 4 × 80 mm IN.PACT Admiral (Medtronic) (Figure 2(b)). In addition, we performed DCB angioplasty for proximal anastomosis with a 6 × 80 mm IN.PACT Admiral (Medtronic) and distal anastomosis with a 5 × 40 mm IN.PACT Admiral (Medtronic) after plain balloon angioplasty with a 5 × 40 mm JADE (OrbusNeich) (Figure 2(c)). The final angiography showed residual stenosis at the proximal anastomosis, and good blood flow was observed (Figures 3(a)–3(e)). IVUS using Eagle Eye Platinum ST (Philips, Amsterdam, Netherlands) showed residual stenosis at the proximal anastomosis with isoechoic plaque and drug-applied findings (Figure 3(f)). Subsequently, prasugrel (3.75 mg/day) and rivaroxaban (10 mg/day) were administered.

**Figure 4:**
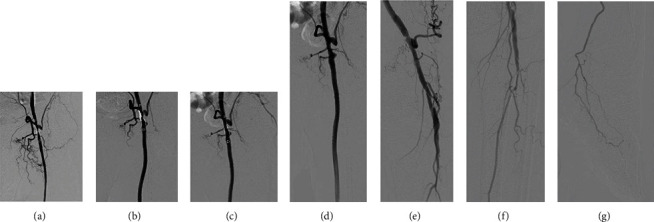
QVA of follow-up angiographies. (a) QVA of immediately after treatment. (b) QVA of 6 months after treatment. (c) QVA of 18 months after treatment. (d–g) Follow-up angiographies 18 months after treatment. QVA: quantitative vessel angiography analysis.

At 6, 12, and 18 months after the procedure, follow-up angiography was performed to confirm the patency of this treatment and did not show restenosis or reocclusion of the treated lesions. Follow-up angiographies at 6 and 18 months showed enlargement of residual stenosis at the proximal anastomosis, which was initially observed immediately after treatment. In quantitative vessel angiography analysis (QVA) for residual stenosis of the proximal anastomosis, the stenosis rates immediately after EVT, at 6 months, and at 18 months were 61%, 51%, and 15%, respectively (Figures 4(a)–4(c)). Lower extremity angiography 18 months after EVT showed good blood flow in the FPB, PA, and PTA (Figures 4(d)–4(g)). Based on this finding, the antithrombotic agent was changed to rivaroxaban (10 mg/day) 18 months after EVT.

## 3. Discussion

Occlusions of the FPB are known to increase the risk of major amputation of the lower extremities [6, 9]. Therefore, it is necessary to maintain patency as much as possible with appropriate surgical or endovascular intervention for FPB failure. Treatment using cutting balloons, self-expanding stents, and drug-coating balloons for stenosis or occlusion of the FPB using the saphenous vein has been reported in a number of cases, with acceptable results [10–12]. However, there are few reports on the treatment options for PFPBO.

The mechanism underlying FPB graft occlusion is stenosis at the anastomosis site caused by intimal hyperplasia [13]. In this case, the cause of graft occlusion was thought to be stenosis of the proximal anastomosis. In cases of PFPBO, abundant thrombus is present in grafts, with a potential risk of embolization. Hence, challenges in the treatment of PFPBO include anastomotic stenosis and removal of a significant thrombus.

Fujimura et al. suggested implanting a Viabahn stent graft in occluded prosthetic grafts [14]. In this study, the primary patency rate at 12 months for PFPBO treatment with the Viabahn stent graft was 92.9%. However, if the distal anastomosis site is covered, the Viabahn stent graft must be implanted up to the popliteal artery, potentially sacrificing critical collateral arteries and eliminating EVT as a treatment option for native SFA. This might be an important issue in developing a treatment strategy since the efficacy of EVT for native SFA in cases of FPB failure has also been reported [15]. In addition, treatment with the Viabahn stent graft requires a good run-off to obtain long-term patency [16].

Another study reported treatment using suction thrombectomy [17]. This method is minimally invasive and effective because it can be considered as an endovascular intervention; an additional stent was implanted as an adjunctive procedure in 83.3% of the cases.

Our new treatment strategy using Fogarty thrombectomy and DCB angioplasty has the potential to remove significant thrombi and treat anastomotic intimal hyperplasia without the use of implantation devices. Thrombectomy using a Fogarty catheter may reduce provisional stenting for anastomosis due to residual thrombus and enable safer DCB angioplasty compared to conventional treatment options. In the present case, graft patency at 18 months was confirmed using duplex ultrasound and follow-up angiographies. Immediately after revascularization, the patient had more than 60% residual stenosis at the proximal anastomosis of the prosthetic bypass graft. Given the patient's history, this lesion was considered a risk for reocclusion of the bypass graft. As previously described, PFPBO has the potential to progress into a limb-threatening condition. Thus, this lesion was evaluated with follow-up angiography and QVA in addition to a duplex ultrasound scan to determine a more optimal treatment strategy. Remarkably, follow-up angiographies showed late lumen enlargement at the proximal anastomosis, which may have been due to the effect of DCB angioplasty on intimal hyperplasia.

The effect of DCB angioplasty on anastomotic stenosis in FPB remains controversial. Matsuura et al. reported a case in which DCB angioplasty was effective in maintaining long-term patency of anastomotic stenosis caused by intimal hyperplasia of a vein graft [18]. In contrast, Björkman et al. reported that there was no significant difference in the treatment for stenosis of FPB grafts between DCB and standard balloon angioplasty [19]. Delayed stenosis regression after DCB angioplasty for native SFA lesions has been previously reported [20]. In this case, the effect of DCB angioplasty was confirmed in the anastomotic stenosis of the prosthetic graft.

Our patient had undergone percutaneous coronary intervention in the past; thus, the antiplatelet drug prasugrel was administered at a Japanese-adjusted dose (3.75 mg/day) [21]. Additionally, given that direct-acting oral anticoagulants were reported to improve patency in FPB cases, rivaroxaban (10 mg/day) was administered based on the patient's renal function [22].

Based on the findings of the present case, our method can be considered as one of the revascularization options for the treatment of PFPBO. One disadvantage of our method is that it is more invasive than EVT. This issue may be resolved by the use of percutaneous mechanical thrombectomy devices instead of Fogarty thrombectomy devices. However, the former devices are currently unavailable in Japan; if they were to be available, we expect that these cases could be treated less invasively. Lastly, the patient initially presented with severe ischemia in the left lower extremity, which raised concerns about the development of a refractory ischemic wound. During the course of treatment, the run-off arteries supplying blood to the affected area may become impaired, potentially leading to further damage to the tissues. In such cases, transcatheter arterialization of the deep veins could be considered to promote the healing of an ischemic wound [23].

## 4. Conclusion

Combination therapy of surgical Fogarty thrombectomy and DCB angioplasty for PFPBO revealed initial procedural success and long-term patency. The therapeutic effect of DCB angioplasty on FPB lesions is controversial, but to the best of our knowledge, there are no reports confirming long-term patency on follow-up lower extremity angiographies. Furthermore, in this case, the effect of the DCB on anastomotic stenosis of the prosthetic graft was confirmed. This revascularization strategy could be a viable treatment option for PFPBO.

## Data Availability

Upon reasonable request, the derived data supporting the findings of this study are available from the corresponding author.

## Abbreviations

PFPBO: Prosthetic femoropopliteal bypass occlusion
FPB: Femoropopliteal bypass
SFA: Superficial femoral artery
CFA: Common femoral artery
EVT: Endovascular therapy
CLTI: Chronic limb-threatening ischemia
DCB: Drug-coated balloon
SFA: Superficial femoral artery
DFA: Deep femoral artery
PA: Popliteal artery
PTA: Posterior tibial artery
IVUS: Intravascular ultrasound
QVA: Quantitative vessel angiography analysis.
